# Supplementary material for: Pleobot: a modular robotic solution for metachronal swimming
Source: Sci Rep. 2023 Jun 13;13:9574. doi: 10.1038/s41598-023-36185-2 (PMC10264458; doi:10.1038/s41598-023-36185-2)
Supplement: Supplementary file 1 — Supplementary Figures. [file 41598_2023_36185_MOESM1_ESM.pdf]

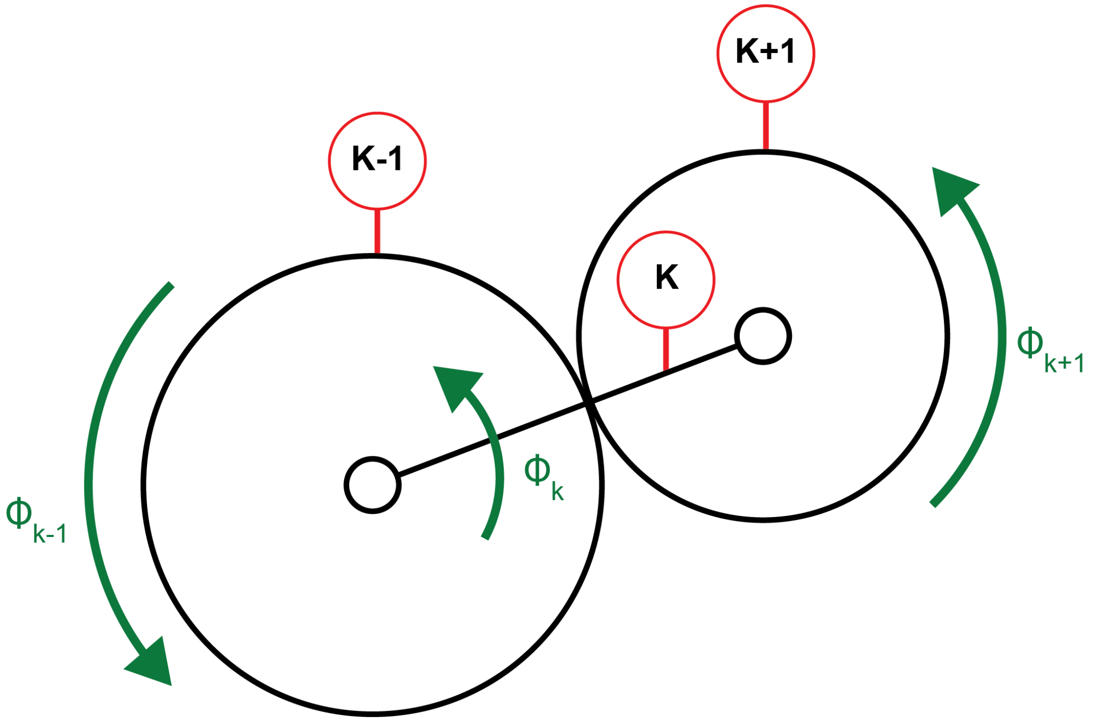

Supplementary figure 1. Epicyclic gear pair. Gear pair with input rotation ϕ and link number k. We obtain the relationship between the driving and driven gears, $\Delta\phi_{k+1}$ and $\Delta\phi_{k-1}$, respectively, and the link connecting both, $\Delta\phi_k$. Image adapted from[1]

References

1. Sandor, G. N. *et al.* Kinematic synthesis of geared linkages. *J. Mech.* **5**, 59–87 (1970).

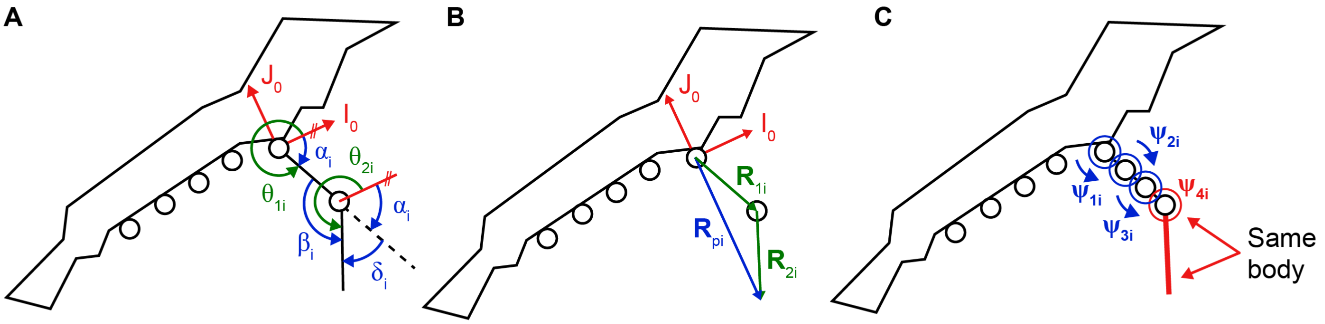

Supplementary figure 2. The locomotive system of the *Pleobot*. Panel A shows a diagram that includes the global reference frame of one pleopod, I_o and J_o, along with angles α_i, the angle between the body axis and the proximal appendage as measured in krill, β_i, the angle between the proximal and distal segment as measured in krill, $\theta_{1i} = 2\pi - \alpha_i$, $\theta_{2i} = \theta_{1i} - \delta_i = \pi + \beta_i - \alpha_i$, and $\delta_i = \pi - \beta_i$. Panel B shows the position of the pleopod, $\mathbf{R}_{pi}$, which is $\mathbf{R}_{pi} = \mathbf{R}_{1i} + \mathbf{R}_{2i}$, where $\mathbf{R}_{1i} = \mathbf{R}(\theta_{1i})\mathbf{r}_{1i}$ and $\mathbf{R}_{2i} = \mathbf{R}(\theta_{2i})\mathbf{r}_{2i}$. Here $\mathbf{r}_{1i}$ and $\mathbf{r}_{2i}$ represent the local reference frame vectors, and $\mathbf{R}(\theta_{1i})$ and $\mathbf{R}(\theta_{2i})$ are the global rotation matrices for the protopodite and endopodite, respectively. Panel C shows angle ψ, the rotation of the gear along link 1 (endopodite), where $\Delta\theta_{1i} = \theta_{1i} - \theta_{1i,0}$, $\Delta\psi_{1i} = \psi_{1i} - \psi_{1i,0}$, $\Delta\psi_{2i} = \psi_{2i} - \psi_{2i,0}$, $\Delta\psi_{3i} = \psi_{3i} - \psi_{3i,0}$, $\Delta\psi_{4i} = \Delta\theta_{2i}$, and $\Delta\theta_{2i} = \theta_{2i} - \theta_{2i,0}$.

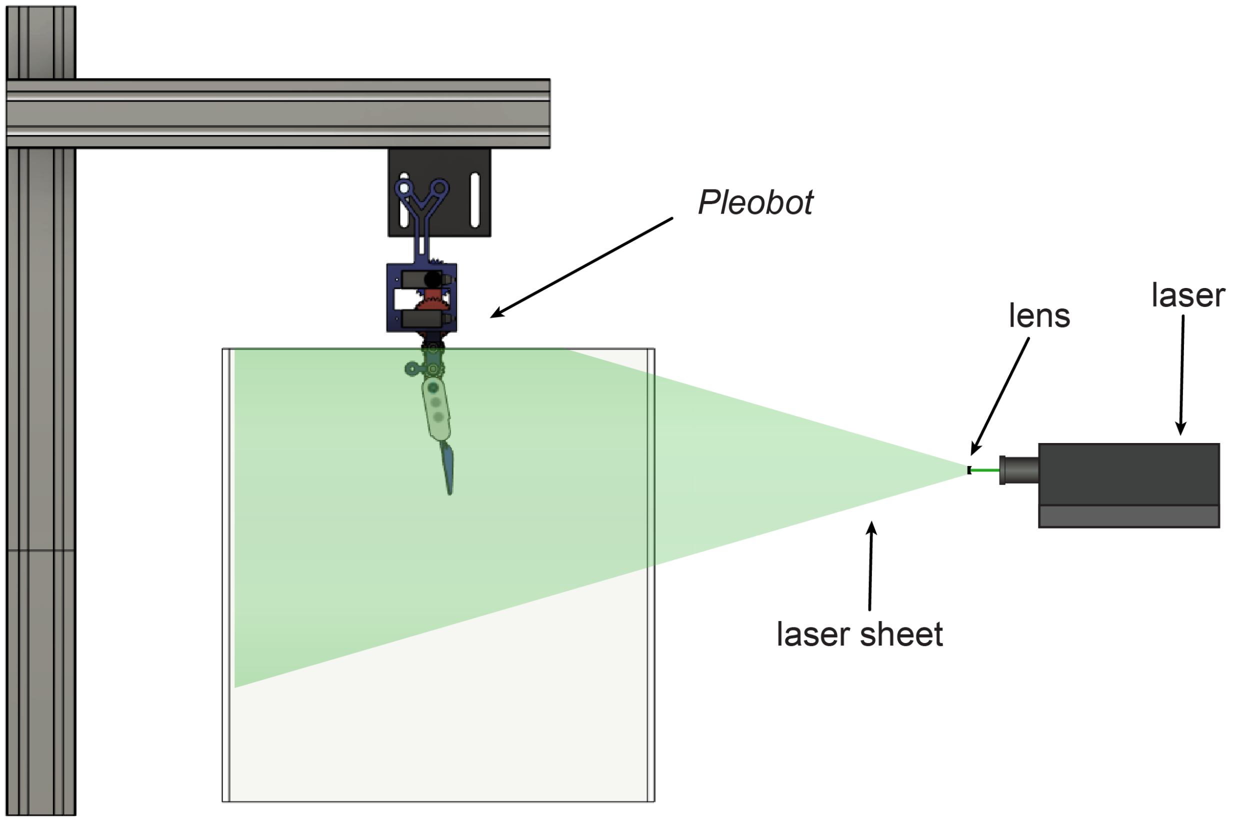

Supplementary figure 3. The flow field around the beating appendage was measured using 2D PIV. The experiments were carried out in a cubic tank of 30 cm by side using a high-speed camera (FASTCAM MINI WX, Photron, 2048 pixels x 2048 pixels) at 125 frames per second, a Nikon lens (Nikon AF-S VR Micro-NIKKOR 50 mm), and a continuous laser (Laserglow, 1 W at 532 nm) with a cylindrical lens to create a laser sheet. The flow was seeded with 10 μm particles (Dantec Dynamics, Skovlunde, Denmark).

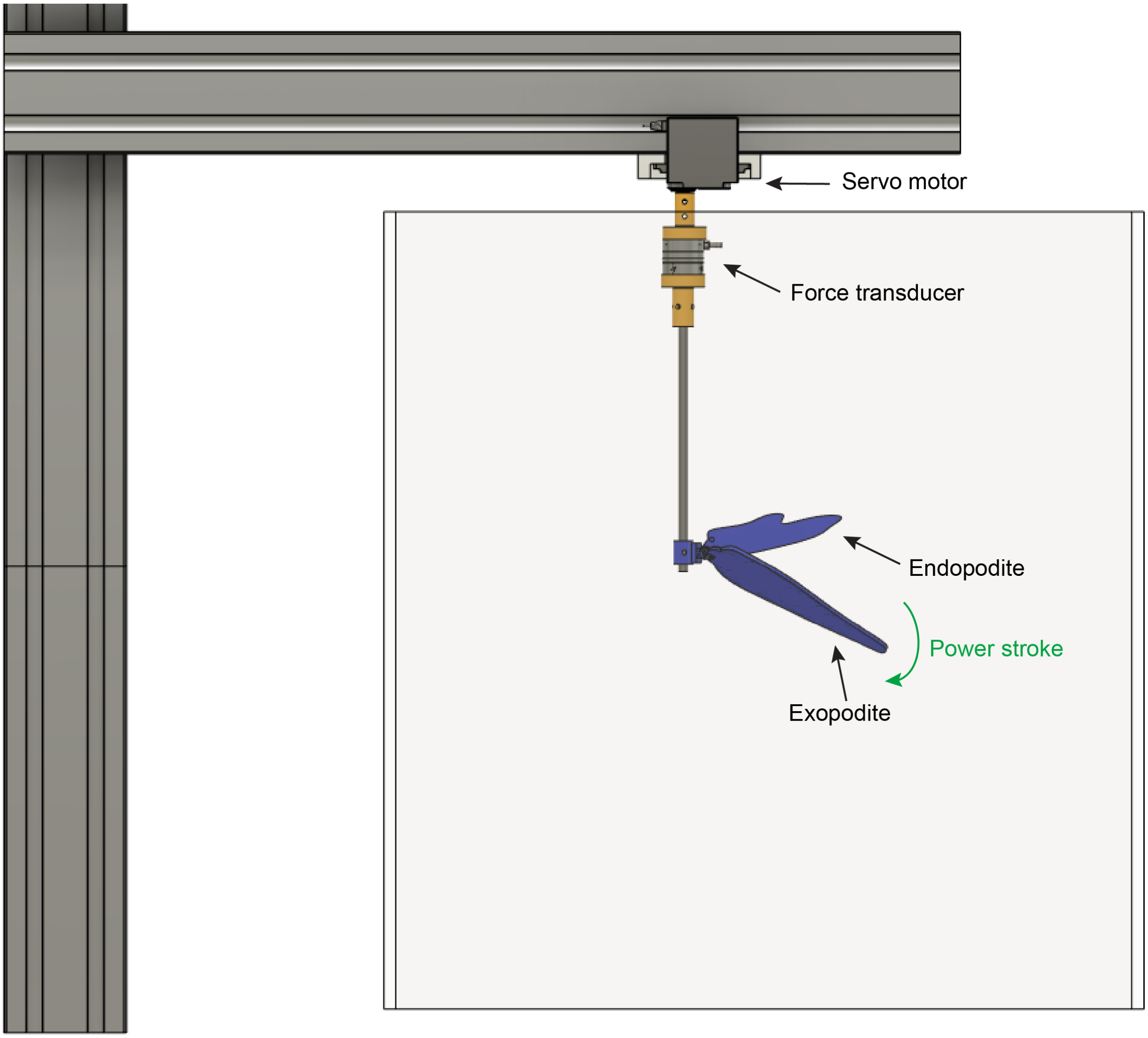

Supplementary figure 4. Experimental setup for force measurements. The force measurements were conducted using a scaled-up (20x) model of the distal appendage (endopodite and exopodite). This was mounted onto a 6-axis force transducer (Nano 17 F/T transducer, ATI), set up in a cubic tank of 30 cm by side. The kinematics were prescribed by the angle Ψ, calculated as $\alpha + 180^o - \beta$. The Re (1500) was used to dynamically scale the experiments by decreasing the beating frequency and increasing the fluid viscosity. A glycerin-water mixture (60% glycerin and 40% water) was used with a dynamic viscosity of 9 cSt, measured with a standard rheometer (Ares-G2, TA Instruments). The appendage was designed to be neutrally buoyant in the water-glycerin mixture.

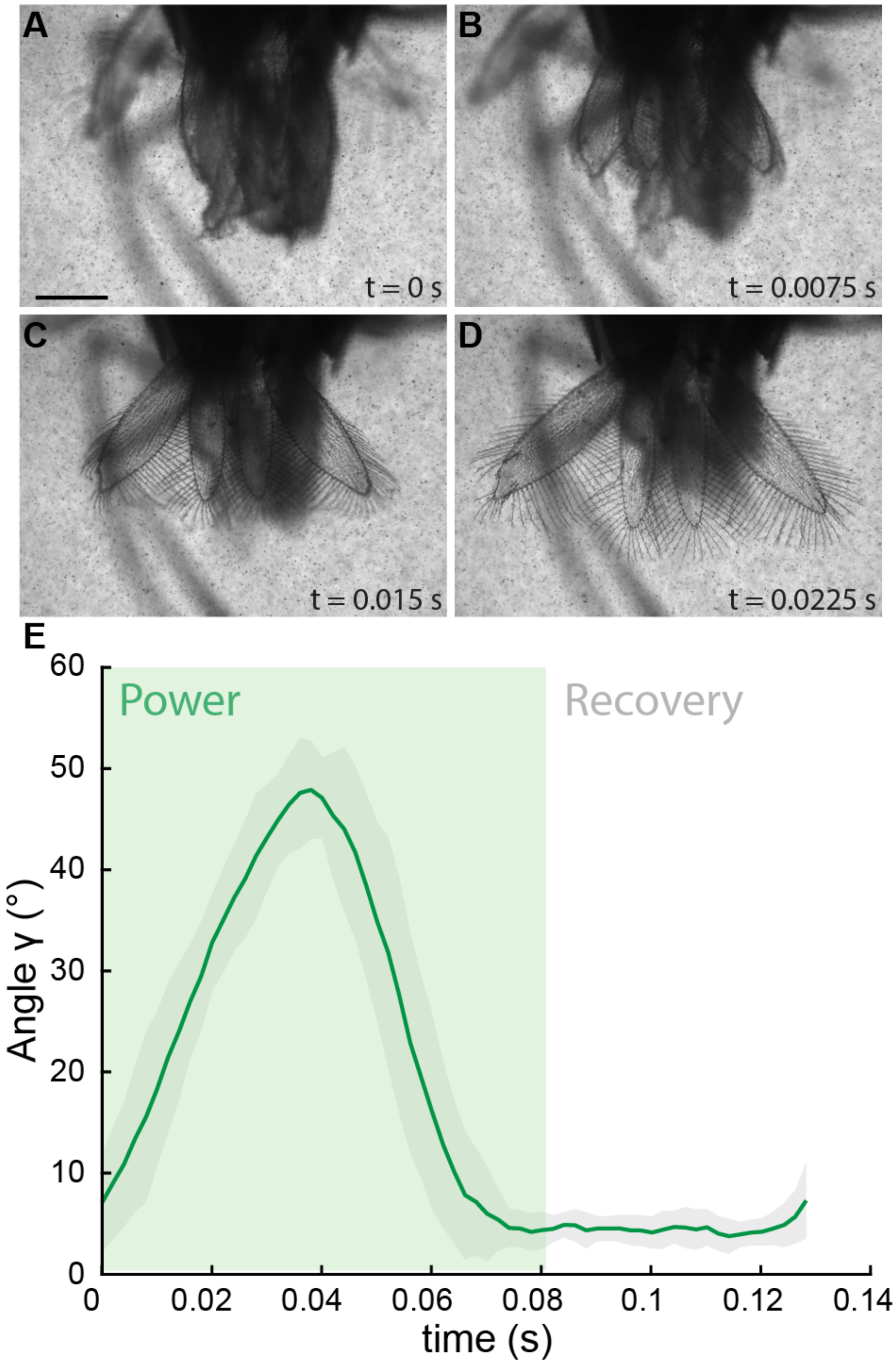

Supplementary figure 5. Characterization of γ in shrimp. The angle between the endopodite and exopodite of a shrimp (*Palaemonetes paludosus*) was measured throughout n = 4 cycles, where the shaded grey region indicates the standard deviation. Panel A-D show the posterior view of the shrimp distal appendages from which γ is measured. Time stamps on panels A-D correspond to the times in panel E. Pleobot shows a similar trend to *P. paludosus* in the way the γ angle evolves during the power and return stroke. The power stroke corresponds to the green shaded area and is labeled as such. The black color bar represents 1 mm.
